# Thermal response of triathletes to 14°C swim with and without wetsuits

**DOI:** 10.1186/2046-7648-4-S1-A49

**Published:** 2015-09-14

**Authors:** Jane Hall, Mitch Lomax, Heather C Massey, Michael J Tipton

**Affiliations:** 1 Extreme Environments Laboratory, Department of Sport and Exercise Science, University of Portsmouth, Portsmouth, UK

## Introduction

Wetsuit use is permitted in Olympic-distance triathlons below a water temperature of 22°C, or 20°C for elite athletes, and mandatory in water below 14°C [1]. They improve insulation against heat loss during swimming, increase buoyancy and reduce the afterdrop (continuing fall in deep body temperature after leaving the water) during subsequent cycling [2]. The aim of this study was to compare performance and body temperature of high-level triathletes swimming in water at 14°C with and without wetsuits, before cycling at race pace for an Olympic-distance event, as the Olympic event is the focus of the International Triathlon Union and International Olympic Committee.

## Methods

12 highly-trained lean (mean sum of 7 skinfolds = 50.15 +/- 22.9 mm) triathletes (10 male, 2 female) aged 15 to 61 years carried out a (self-paced) front crawl race-pace 20 minute swim (the approximate time for elite triathletes to complete the swim in an Olympic-distance event) in a flume at 14°C, followed by a transition and race-pace cycle on a stationary bicycle until deep body temperature (T_re_) had returned to its starting point. T_re _was monitored with a rectal thermistor, inserted to 15 cm past the anus. 10 athletes completed the swim without wetsuits (skins swim, 'SS') and 9 athletes completed the swim wearing their own triathlon-specific wetsuits (wetsuit swim, 'WS'). 7 males completed both swims. Air temperature was maintained at 12°C, with fans providing wind speed during the cycle of at least 15 km.h^-1^. The rate of fall of T_re _was calculated from the point at which the fall became linear, and data for those who completed both swims was analysed by paired samples T-tests (n = 7).

## Results

Four athletes out of ten were unable to complete the SS condition. All athletes completed the WS condition. There was a significant difference (p < 0.05) between SS and WS in linear rate of change of T_re _in the swim (SS = -0.076°C.min^-1 ^or -4.56°C.hr^-1^; WS=-0.023°C.min^-1 ^or -1.38°C.hr^-1^), total change in T_re _in the swim (SS=-1.12°C; WS=-0.21°C), afterdrop on the bike (SS = 0.31°C; WS = 0.02°C) and time taken on the bike for T_re _to return to starting point (SS = 54.57 mins; WS = 10.56 mins). During SS, cooling typically continued at the same linear rate during transition and the early part of the cycle (10.6 mins), while in WS, athletes started to rewarm almost immediately on starting the cycle (1.4 mins) with little or no afterdrop.

## Discussion

In SS, the rate of cooling during the swim was rapid although swimmers were able to maintain speed. During cycling, a convective afterdrop pattern was observed. Wearing a wetsuit reduced the fall in T_re_, and also kept skin temperatures higher, allowing athletes to rewarm on the bike with little or no afterdrop.

## Conclusion

Purposely designed triathlon wetsuits help protect T_re _for lean, high-level athletes racing in 14°C water, as well as reducing or removing subsequent afterdrop while cycling. Whether this has an impact on performance on the bike remains to be seen.
